# A Review on Green Composites Based on Natural Fiber-Reinforced Polybutylene Succinate (PBS)

**DOI:** 10.3390/polym13081200

**Published:** 2021-04-08

**Authors:** Mokgaotsa J. Mochane, Sifiso I. Magagula, Jeremia S. Sefadi, Teboho C. Mokhena

**Affiliations:** 1 Department of Life Sciences, Central University of Technology, Private Bag X20539, Bloemfontein 9300, South Africa; sifisom61@gmail.com; 2 Department of Physical and Earth Sciences, Sol Plaatje University, Kimberley 8301, South Africa; 3 Department of Chemical, Metallurgical and Materials Engineering (Polymer Technology Division), Institute of Nano Engineering Research (INER), Tshwane University of Technology, Pretoria 0001, South Africa; mokhenateboho@gmail.com

**Keywords:** natural fiber, green composites, surface modification, polybutylene succinate, biodegradable

## Abstract

The need for utilization of environmentally friendly materials has emerged due to environmental pollution that is caused by non-biodegradable materials. The usage of non-biodegradable plastics has increased in the past decades in many industries, and, as a result, the generation of non-biodegradable plastic wastes has also increased. To solve the problem of non-biodegradable plastic wastes, there is need for fabrication of bio-based polymers to replace petroleum-based polymers and provide strategic plans to reduce the production cost of bioplastics. One of the emerging bioplastics in the market is poly (butylene succinate) (PBS) and it has been the biopolymer of choice due to its biodegradability and environmental friendliness. However, there are some disadvantages associated with PBS such as high cost, low gas barrier properties, and softness. To lower the cost of PBS and enhance its properties, natural lignocellulosic fibers are incorporated into the PBS matrix, to form environmentally friendly composites. Natural fiber-based biocomposites have emerged as materials of interest in important industries such as packaging, automobile, and construction. The bonding between the PBS and natural fibers is weak, which is a major problem for advanced applications of this system. As a result, this review paper discusses various methods that are employed for surface modification of the Fibers The paper provides an in-depth discussion on the preparation, modification, and morphology of the natural fiber-reinforced polybutylene succinate biocomposites. Furthermore, because the preparation as well as the modification of the fiber-reinforced biocomposites have an influence on the mechanical properties of the biocomposites, mechanical properties of the biocomposites are also discussed. The applications of the natural fiber/PBS biocomposites for different systems are also reported.

## 1. Introduction

**Figure 1:**
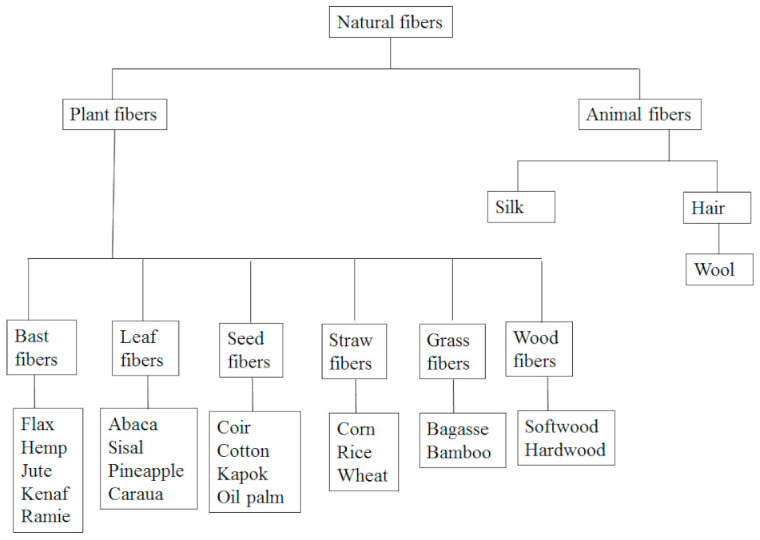
Classification of natural Fibers.

Plastics play a huge part in our daily routine, which has caused the worldwide production and disposal of plastics to increase extensively in the last couple of years due to their applications in different industries [1]. The kind of waste that emanates from polymers is normally bulkier than that of organic materials; as a result, a large portion of this type of waste does not degrade naturally. Due to the continuous demand for non-biodegradable polymers and their composites, there has been significant waste resulting from the non-biodegradable materials that accumulate in landfills, as a result occupying a huge space and initiating a serious challenge in terms of environmental impact. Due to the occupation of the landfill, most non-biodegradable polymer wastes are incinerated [2]. Incineration has serious drawbacks including high cost and the release of harmful gases which might be harmful to the environment. Based on the above dangers of non-biodegradable polymers disposal, it is urged that new effective environmentally friendly and biodegradable materials are required for advanced applications. There is a strong interest globally in the fabrication of biocomposites, which are known as “green composites”, due to the current demand of developing materials with circulating resources. There is more public environmental awareness that has forced industrial manufacturers to make an effort in terms of producing environmentally friendly materials [3]. The solution to a non-polluted environment would be the utilization of green biopolymer composites. Green composites are defined as those composites that are fabricated from a biopolymer matrix in combination with a natural fiber. The well-known biopolymer matrices are: polybutylene succinate (PBS) [4], poly hydroxyalkanotes (PHA) [5], polylactic acid (PLA) [6], poly (poly(ε-caprolactone) (PCL) [7] and thermoplastic starch [8]. Due to their biocompatibility, biodegradability and environmental friendliness, these biopolymers have been used for various applications [9]. The natural fibers include ramie, sisal, coir, hemp, etc. The whole idea of preparing natural fiber green composites is to ensure that the resultant material is eco-friendly and cost comparable when compared with conventional materials. One well-known biopolymer is poly(butylene succinate) (PBS) which is generally distinguished by properties such as good processing, and excellent chemical and thermal resistance. However, there are some limitations of the PBS which hinder the practical application of this polymer, such as high cost, low gas barrier properties, and softness [10]. To decrease the high cost of PBS and enhance its properties, PBS is normally reinforced with natural Fibers Natural fibers are used as reinforcing fillers due to their advantages such as low density and low cost, as well as being environmentally friendly and biodegradable [3,11,12,13,14,15]. Natural fibers are defined as those fibers which are not synthesized but produced from sources such as plants and animals [16,17,18]. Figure 1 illustrates the classification of different natural Fibers Table 1 illustrates the world production per year of selected natural Fibers.

Natural fiber-reinforced polymer composites have been the composites of interest in various applications including automobiles, food packaging, aeroplane interiors, storage devices and building-related applications [3,20]. The utilization of biopolymer matrices in the fabrication of green composites aims to reduce the dependence on petroleum resources. Another important factor about biopolymers is their ease in fabrication with different processing techniques (viz compression molding, injection molding, extrusion, and melt mixer) in the formation of biocomposites. As a result, the fabrication of biocomposites with high performance is essential. A lot of effort [4,10,21,22,23] has been dedicated to the fabrication of PBS/natural fiber biocomposites. Various natural lignocellulosic fibers such as ramie [21], jute fiber [4], bamboo fiber [22], and sisal fiber [23], to mention a few, have been added into the PBS matrix in order to improve the properties of the resultant biocomposites. This review paper discusses the preparation, morphology, modification, and mechanical properties of PBS/natural fiber biocomposites.

## 2. PBS Synthesis, Structure, and Properties

PBS is an aliphatic, biodegradable and bio-based polyester. PBS belongs to a group of biodegradable polymers exhibiting properties such as excellent biodegradability, thermoplastic processibility and balanced mechanical properties [24]. It looks like a white thermoplastic polymer exhibiting a density of 1.25 g/cm^3^, a glass transition temperature range of −45 °C to −10 °C and a melting temperature range of 90 °C to 120 °C [25]. The tensile strength of unoriented PBS specimens can reach up to 30–35 MPa, which is comparable to that of polypropylene and polyethylene [24] (see Table 1). PBS is also a flexible polyester with a Young’s modulus in the range of 300–500 MPa depending on the degree of crystallinity [24]. PBS also has a wide temperature window for thermoplastic processing [24]. It can be processed similarly to polyolefins in the range of 160 °C to 200 °C under controlled conditions [25]. Therefore, this qualifies the resin for processing methods such as extrusion, injection molding, thermoforming and film blowing [24]. However, PBS has an advantage over polyolefins due to its excellent biodegradability, which makes it an attractive alternative to other non-renewable polymers [25]. The physical properties of PBS as compared to those polyolefins are shown in Table 2.

**Figure 2:**
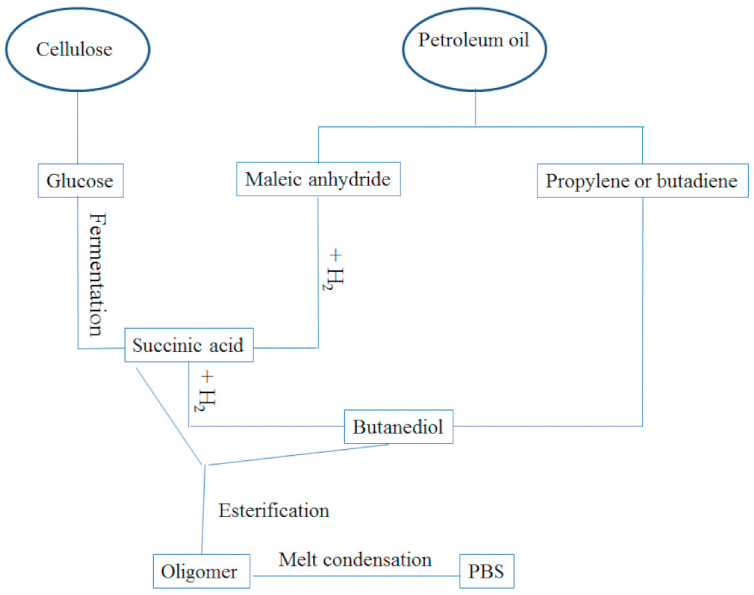
Flow chart representing the production of PBS.

**Figure 3:**
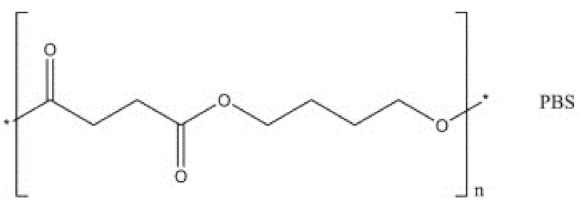
Chemical structure of polybutylene succinate (PBS) [43]. Copyright obtained from Elsevier.

PBS is synthesized via the polycondensation of succinic acid (or dimethyl succinate) and 1,4-butanediol (BDO). The succinic acid and BDO monomers are derived from either fossil-based or renewable resources [24], as shown in Figure 2. Figure 2 is an illustration of the flow chart representing the production of PBS and Figure 3 is an illustration of the general structure of PBS. Furthermore, the physical properties and biodegradation of PBS can be varied widely by copolymerizing it with different types and various contents of monomers [24]. The different types of comonomer units used in copolymerization, including adipic acid [26,27,28], terephthalic acid [29,30,31], methyl succinic acid [32,33,34], 2,2-dimethylsuccinic acid [35], benzyl succinic acid [36], ethylene glycol [36,37,38] and 1,3-propanediol [39,40,41]. Furthermore, PBS is used in a wide range of applications such as in packaging films, agriculture mulch films, packaging materials, vegetation nets and compost bags [42], to mention just a few. However, the poor tensile properties, low melt viscosity and gas barrier properties are some of the major drawbacks that limit the applications of PBS [42]. Therefore, the incorporation of fillers into the PBS matrix is one of the effect ways of improving the properties of PBS, especially its mechanical properties [42].

## 3. History of Natural Fibers

**Figure 4:**
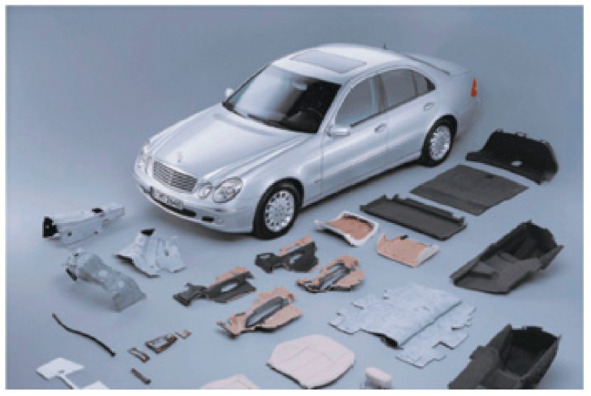
Automobile components fabricated with natural fiber related composites. Reprinted with permission from the publisher [16]. Open access.

Natural fibers have a long, proud service history to the human race, having been used since prehistoric times [44]. Their use can be traced back more than 10,000 years [45]. As early as 8000 BC, natural fibers were already used as textiles in the Middle East and China [45]. For instance, clothes made from flax fibers were already available as early as 3000 BC [46]. The Babylonians used flax fiber for burial purposes as early as 650 BC [46]. Therefore, it has been argued that flax is the oldest fiber used by mankind [47]. Textile fibers have been used to make clothes for the last 4000 or 5000 years [47]. Since prehistoric times, natural fibers such as flax, hemp, silk, wool and cotton have been the only fibers used until 1885, when the first artificial or man-made fibers were introduced into the market [47]. Furthermore, the idea of using plant fibers as reinforcing materials can be traced back to prehistoric times when straw was incorporated into bricks during the Pharaonic period [45]. Similar plant fiber-based pottery was also made by Inca and Mapa civilizations [45]. In the 1930s, Henry Ford used hemp to construct an entire automobile body [45]. Presently, German automobile manufacturers such as BMW and Mercedes have also begun to utilize natural fiber-reinforced polymer composites in various automobile parts [45]. Figure 4 illustrates the utilization of natural fibers in different parts of the Mercedes Benz automobile. Mercedes utilizes coconut fiber-reinforced rubber latex composites in the seats of their Benz E-class model [16]. Daimler-Benz also utilizes various natural fibers (such as sisal, jute, coconut, European hemp and flax) as reinforcing materials in high quality polypropylene components in order to replace glass fibers [16]. Furthermore, Daimler-Benz has also been developing their dashboards, centre armrests consoles, seat shells and the panelling on their seat backs by increasing their utilization of natural fiber-reinforced polymer composites by about 98% when compared to previous models [16]. The natural fiber-reinforced polymer composites used in their development were based on natural fibers such as abaca and flax as reinforcing materials [16].

The BMW group uses a lot of natural fiber-reinforced composites in their automobiles as well. In 2004 alone, the BMW group utilized about 10,000 tonnes of natural fiber in their automobiles [16]. Each BMW 7 series car is made up of about 24 kg of renewable raw materials, with flax and sisal used to make the interior door lining panels of the car [16]. BMW also uses cotton in their soundproofing, wool in their upholstery and wood fiber in their seat backs [16].

## 4. Structure of Natural Fibers

**Figure 5:**
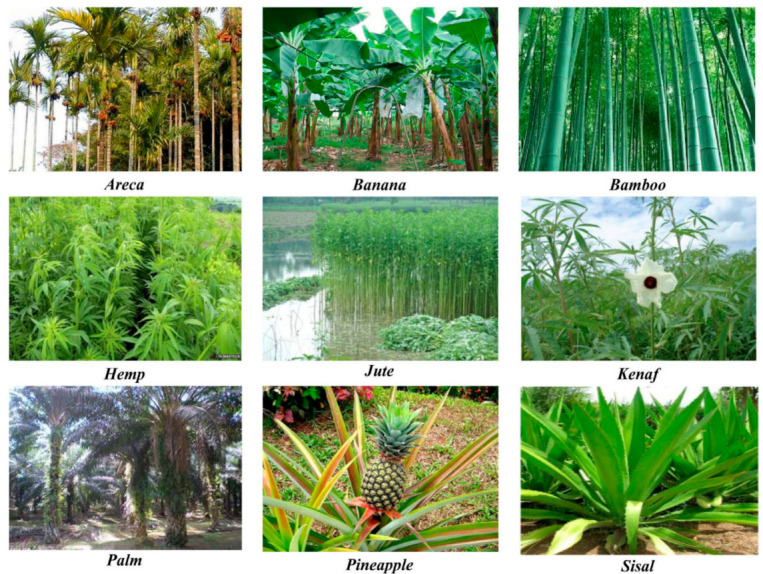
Types of natural plant Fibers Reprinted with permission from the publisher [50]. Open access.

**Figure 6:**
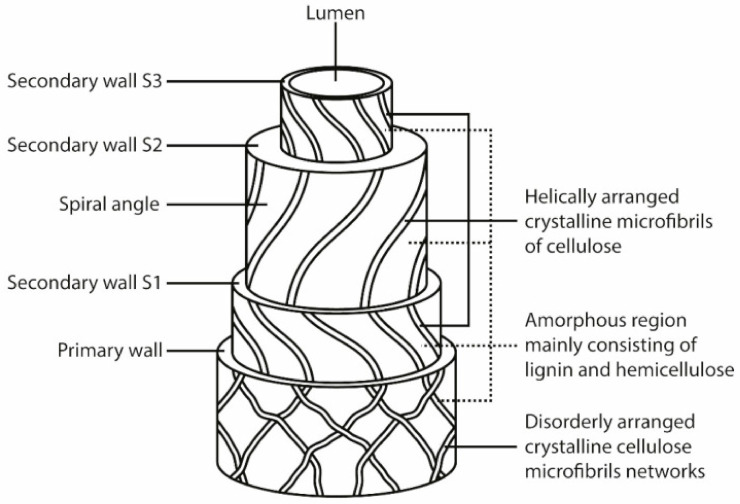
General structure of a natural fiber. Reprinted with permission from Elsevier [51].

One natural plant fiber is like a unit cell that is 1 mm to 50 mm in length and approximately 10 μm to 50 μm in diameter [48]. Figure 5 shows the different natural plant fibers available in nature. Natural fibers look like microscopic tubes which consist of cell walls surrounding a central lumen [48]. The central lumen controls the water uptake abilities of the fiber [48]. Each cell wall of the natural fiber consists of oriented cellulose microfibril reinforcements, which are semi-crystalline, incorporated into a matrix of hemicellulose and lignin [48], as shown in Figure 6. The cellulose microfibrils are approximately 10 nm to 30 nm in diameter and are composed of 30–60 molecules of cellulose linked together in a chain-like succession [48,49]. In addition to providing rigidity, cellulose fibrils also enhance fiber mechanical characteristics such as the tensile and flexural strengths [49].

The cellulose microfibrils are cemented together by the molecules of the hemicellulose matrix [48]. This is because the molecules of the cell wall hemicellulose have the ability to form hydrogen bonds with cellulose [48]. This allows the hemicellulose molecules to create a network of cellulose/hemicellulose structures, which are thought to be the main components in the fiber cell [48]. The strength of the cellulose/hemicellulose structures is improved by the cementing effect of the lignin matrix, which is hydrophobic [48]. The lignin matrix also forms a protective covering that protects the internal components of the fibers against decomposition by microbes [52].

The cell walls of natural fibers are composed of a primary cell wall and a secondary cell wall [48,53], as shown in Figure 6. The primary cell wall is composed of closely packed, unattached and irregularly arranged cellulose microfibrils [48]. Contrastingly, the secondary cell wall is composed of three discrete layers—the outer layer (S1), middle layer (S2) and inner layer (S3 [48]. The S2 layer is the thickest and the most significant in determining the mechanical performance of the fibers [48]. The cell walls of natural fibers have different compositions. For instance, the cellulose and lignin–hemicellulose matrix ratio as well as the spiral angle of cellulose microfibrils are different for each cell wall [54]. The spiral angle is the angle between the helical spirals of microfibrillar cellulose and the fiber axis [48]. The spiral angle (microfibrillar angle) differs from fiber to fiber [48]. Furthermore, the mechanical characteristics of a fiber depend on the content of cellulose, the spiral angle as well as the degree of polymerization [48]. For instance, fibers consisting of an increased content of cellulose and degree of polymerization, as well as a lower spiral angle, exhibit an enhanced tensile strength and modulus [48]. The degree of polymerization depends on the origin of the fibers [48]. Furthermore, cellulosic fibers or natural fibers are composed of two phases; an amorphous and a crystalline domain [48]. The crystalline domain has a high degree of organization whilst the amorphous domain has a low degree of organization [48]. A continuous removal of the amorphous domain leads to the appearance of fibrils with a high crystallinity (up to 100%), and whiskers are eventually obtained [48]. The degree of crystallinity is determined by the original nature of the fiber source [48]. Cellulose fibers can either be natural (cotton linter, wood, bamboo, bagasse) or regenerated (viscos and lyocell). Regenerated cellulosic fibers are mode from chemically dissolving natural cellulose fibers after spinning treatment [55]. Natural cellulose fibers such as cotton, sisal, ramie, banana and flax have enhanced degrees of crystallinity (i.e., 65–70%) [48]. However, regenerated cellulose has a crystallinity of only 35–40% [48]. The reinforcing ability of natural fibers is determined by the origin of the cellulose and its degree of crystallinity [56]. The crystallinity of cellulose is partially due to the hydrogen bonds that exist between cellulose chains [54]. However, hydrogen bonds are also found in the amorphous phase even though it is less organized [54]. The water uptake behavior of fibers is influenced by the content of cellulose and hemicellulose in the fiber structure [57]. Cellulose consists of a lot of hydroxyl groups which can form hydrogen bonds with water [48]. The chemical bonding between water and the cellulose hydroxyl groups does not only occur at the surface but also in most of the material [48]. The water uptake of the fiber is determined by the relative atmospheric humidity around the fiber in an equilibrium state [48]. The sorption isotherms of the fibers depend on the pure nature of the cellulose and its crystallinity degree [48]. All the O-H groups of the amorphous phase easily interact with water, whilst few water molecules interact with the O-H groups of the crystalline phase [48].

**Figure 7:**
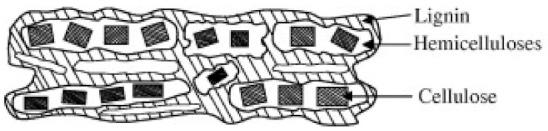
Structural arrangement of the three components of a natural fiber cell wall. Reprinted with permission from Elsevier [53].

**Figure 8:**
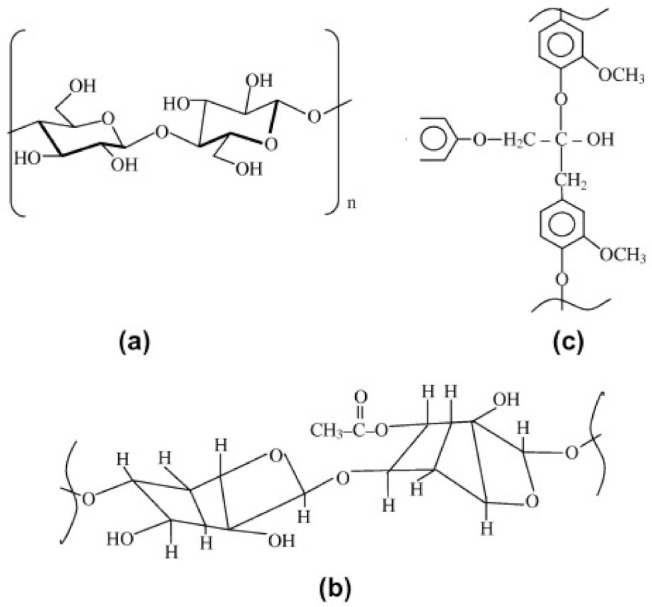
General structures of (**a**) cellulose, (**b**) hemicellulose and (**c**) lignin. Reprinted with permission from Elsevier [53].

Generally, natural fibers mainly consist of hemicellulose, cellulose, pectins, waxes and, lignin [48]. The pectin provides flexibility for the fibers and the waxes form an outer layer on the fibers [51]. Figure 7 and Figure 8 show the arrangement and chemical structures of the three fiber components in the cell walls of natural fibers (lignin, hemicellulose, and cellulose). As illustrated by Figure 8a, cellulose is made up of three hydroxyl groups (OH). Two of them form intramolecular hydrogen bonds in the cellulose macromolecule itself, whilst the other OH groups form intermolecular hydrogen bonds with external cellulose molecules [53,58]. Hemicellulose, which is mainly situated in the primary cell wall, is made up of branched polymers which consist of 5–6 carbon sugars [53] (see Figure 8b). Lignin is amorphous and structurally aromatic [53] (see Figure 8c). Pectin is made up of complex polysaccharides which consist of side chains that are crosslinked with arabinose sugars and calcium ions [53]. Additionally, the fiber structure also consists of inorganic ash components and organic extractives [53]. The organic extractives determine the color, odor and resistance to decay of the fibers, whilst the inorganic constituents are responsible for enhancing the abrasive nature of the fiber [53]. Table 3 shows a summary of the constituents and the amount of each component found in different natural Fibers.

## 5. The Concept of Natural Fiber/Biopolymer Green Composites

**Figure 9:**
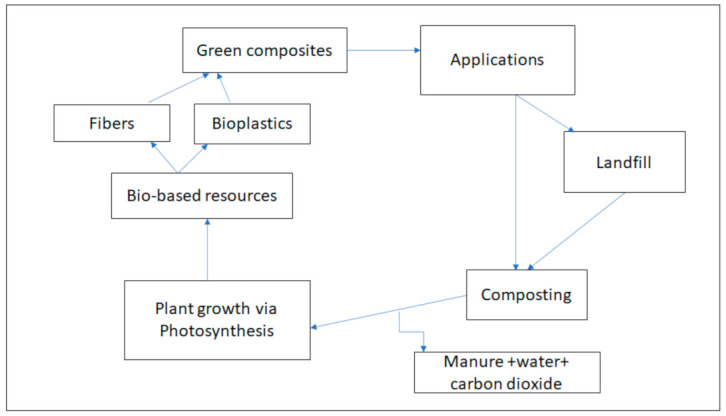
Typical example of the expected life cycle of a green composite.

The concept of green composites is not really a new topic to mankind, yet there are still few composites that are regarded as biocomposites. In the late 1990s, Herrmann and co-workers fabricated a green composite from hemp and ramie fibers, with a matrix blend of starch and polyvinyl alcohol (PVC), and this blend was termed a bio-composite [101]. Since the aim of fabricating green composites is to decrease the carbon footprint in our environment, it is important to understand the anticipated life cycle of green composites, as shown in Figure 9. According to Figure 9, the green composites are expected to release both water and carbon dioxide when degraded by microorganisms; furthermore, the incineration of such composites is expected to release gases that are not harmful.

## 6. Preparation, Modification and Morphology

### Natural Fiber/PBS Biopolymer Composites

**Figure 10:**
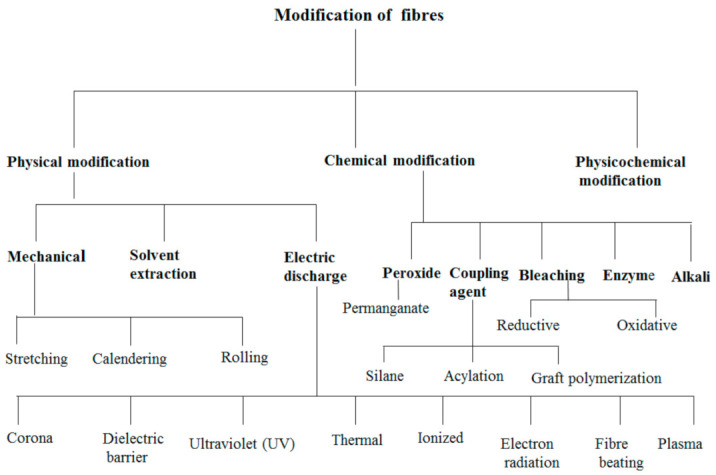
Different methods for fiber modification.

**Figure 11:**
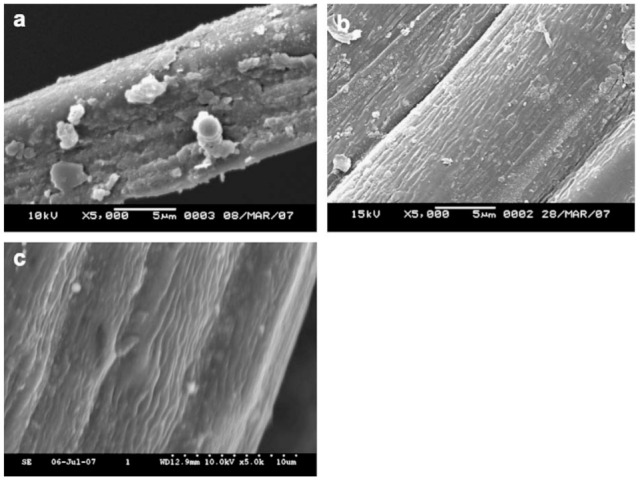
Surface modification of the fiber: (**a**) no modification, (**b**) alkali, and (**c**) coupling treatments. Reprinted with permission from Elsevier [103].

**Figure 12:**
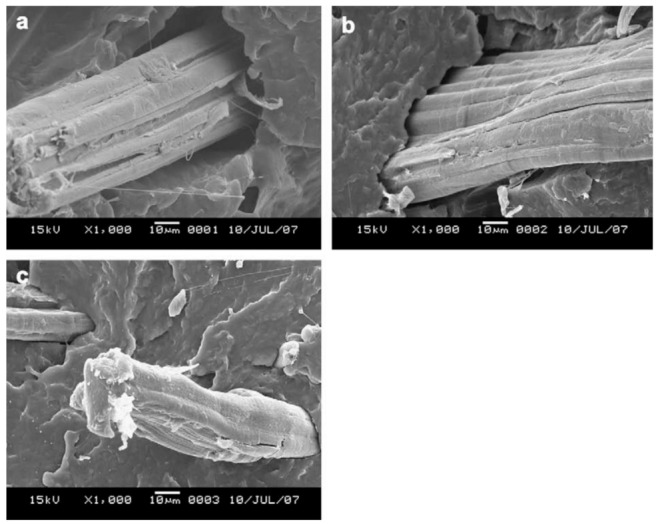
Surface morphology of (**a**) untreated jute/PBS, (**b**) alkali-treated jute fiber/PBS and (**c**) coupling-treated jute fiber/PBS biocomposites. Reprinted with permission from Elsevier [103].

The morphology of natural/PBS biocomposites was found to be a very important aspect of these biocomposites since the resultant morphology had an impact on properties such as mechanical strength and moisture. It was found that the morphology of the natural fiber biocomposites was affected by the type of modification used to modify the natural fiber [102,103,104,105], the content of the fiber [106], and the preparation method [106] of the composites. It is well known from the literature [107,108,109,110,111,112,113,114,115,116,117] that the interfacial interaction between the hydrophobic polymers and hydrophilic fibers is very weak, which consequently affects the properties of the resultant natural fiber/polymer composites. Different processes have been employed to improve the interfacial interaction between the fiber and polymer matrices, including chemical and physical methods [118]. The processes involved in improving interfacial bonding between fibers and polymer matrices may involve the incorporation of a third component (compatibilizer) which has the ability to interact with both the fiber and polymer matrix [119], or to modify the surfaces of fibers [120]. There are a few studies on the method of incorporating compatibilizers into natural fiber-reinforced PBS composites. However, there are a few studies which are based on the incorporation of epoxy (epoxidized linseed oil (ELO) and epoxidized soybean oil (ESBO)), maleic anhydride (maleinized linseed oil (MLO) and dodecenyl succinic anhydride (DDSA)) and acrylic compatibilizers (methyl methacrylate (MMA) and acrylic acid (AA)) into PBS composites reinforced with lignocellulosic fillers such as almond shell [121], lignin [119] and wheat bran [122]. In all these studies, it was shown that the incorporation of the compatibilizer improved the compatibility of the composites. Therefore, such compatibilizers can be considered for use in natural fiber/PBS composites in the future. Furthermore, in most natural fiber-based biocomposites, fiber surface modification seems to be the most commonly used method. Fiber modification is divided into three classifications (Figure 10), i.e., (i) physical, (ii) chemical, and (iii) physicochemical treatments. The aim of fiber treatment is to get rid of impurities, change the crystalline structure, enhance the fiber–matrix interface, and improve the adhesion between the polymer and fiber [60]. Generally, surface modification removes a certain amount of the lignin, hemicellulose and pectin that covers the external cell wall of a fiber [103]. This leaves the fiber cleaner and rougher than before [103]. During chemical treatment of fibers, the hydrophilic nature of natural fibers induced by the pre-dominance of –OH groups in the fiber is weakened and, therefore, the compatibility of the fiber with hydrophobic PBS is enhanced [103]. Bin et al. modified the cotton fiber through the steam explosion method and the modified cotton was incorporated into the PBS matrix [10]. According to Figure 10, modification of fibers using the steam explosion method is classified as a physicochemical modification process. The cotton stalk bast fibers (CSBF)/PBS composites were prepared using a plastic-mixing mill at 170 °C, with a mixing time of 5 min. The mixed samples were subjected to compression molding at 150 °C, with a pressure of 10 MPa. The morphology of the composite was analysed by scanning electron microscopy, and it was reported, with the analysis taken from CSBF/PBS (40/60), that the fiber was well dispersed in the polymer matrix. The comparison between alkali-treated jute fiber and coupling-treated jute fiber-reinforced PBS composites was investigated [103]. Jute fibers were treated with 5% sodium hydroxide solution for alkali treatment and soaked in 1.5% of KH-570 (silane coupling agent) for coupling agent treatment. The surface modification, in this case alkali and coupling agent, removed lignin, impurities and waxes, whereby the fiber became cleaner and rougher (Figure 11), which enables a better interaction between the fiber and the PBS matrix (Figure 12). The mechanism behind the improved interaction between alkali- and coupling agent-treated jute fibers and PBS (Figure 12), is based on reducing the hydroxy groups (OH groups) of the natural fibers, as shown by equation 1 and 2, respectively, whereby the OH groups are converted to alkoxides.

NaOH + Cell-OH → Cell-O^−^Na + H_2_O (1)

(N.B. NaOH = sodium hydroxide, Cell-OH = cellulose fiber, Cell-O^−^Na = cellulose fiber-alkali complex, H_2_O = water)

CH_2_=C(CH_3_) COO(CH_2_)_3_ Si (OCH_3_)_3_ + Cell-OH → CH_2_=C(CH_3_) COO(CH_2_)_3_ Si (OH)_2_O-Cell + H_2_O (2)

(N.B CH_2_=C(CH_3_) COO(CH_2_)_3_ Si (OCH_3_)_3_ = 3-Methacrylpropyltrimethoxysilane (MPMS), Cell-OH = cellulose fiber, MPMS grafted cellulose fiber, H_2_O = water).

**Figure 13:**
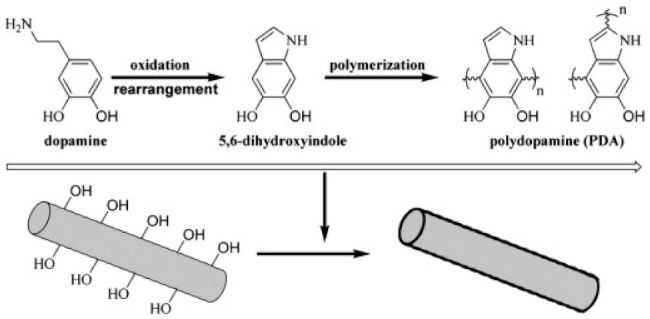
An illustration of the polydopamine (PDA) coating of ramie fiber. Reprinted with permission from Elsevier [121].

The surface modification of the natural fibers has been successful (viz the improvement in interfacial adhesion between the fiber and the polymer) as a result of utilizing different modifiers such as silane, acetylation, alkali, and coupling agents. According to Zhou et al. [123], these modifying agents had a negative impact on the strength and structure of the fiber to some extent. Furthermore, it was mentioned by the authors that the solvents involved in the modification process might be harmful to the environment; as a result, they modified ramie with a harmless modifier in the form of dopamine. According to Figure 10, modification using dopamine is classified as a chemical modification process. The use of dopamine as a modifier for ramie was further influenced by its ability to adhere to materials that are submerged in it. Dopamine underwent oxidation and polymerization to form polydopamine (PDA), which was coated into the surface of the rami fiber (Figure 13). PBS/ramie composites, with 10% natural fiber, were prepared by hot presser. In this study, the interfacial crystallization of the matrix in the form of PBS with dopamine-treated ramie fiber was investigated by utilizing a polarized light microscope. The focus of the study was to provide an alternative and efficient method of enhancing the interfacial interaction between the PBS and ramie through a controlled interfacial crystallization. The addition of non-treated ramie into PBS showed no nucleation on the crystallization of the polymer, while the incorporation of dopamine-treated ramie fiber resulted in trans crystallization, which can enhance the interaction between ramie and PBS.

**Figure 14:**
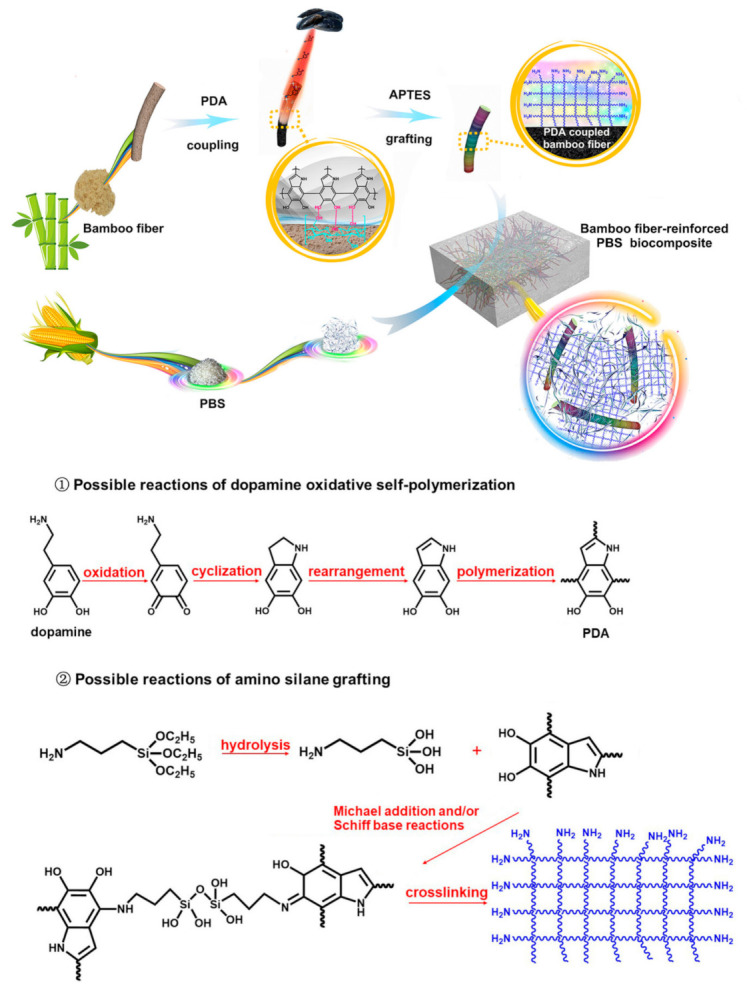
A scheme illustrating the modification of the fiber, a reactive mechanism between modifiers and the fabrication of the biocomposites. Reprinted with permission from Elsevier [124].

Hong et al. [124] modified the bamboo fiber with the synergy of PDA and 3-aminopropy triethoxysilane (APTES) and the modified fiber was incorporated into the PBS matrix. The biocomposites were prepared in three steps: (i) mixing, (ii) extrusion and (iii) compression in hot pressing. A similar method to the compression in hot pressing technique has also been used by other authors [125] before in their preparation of PBS/waste paper (WP) biocomposites. In this study, the contents of the fiber and PDA were kept constant while varying the content of the APTES. The surface modification (viz PDA and APTES) of the bamboo fiber is clearly illustrated in Figure 14. As explained earlier in this document, the formation of PDA occurs through the oxidation and polymerization of dopamine (Figure 14). The morphology of the modified and unmodified bamboo reinforced PBS biocomposites were analysed by field emission scanning electron microscopy. Smooth surfaces were observed for unmodified system, while the synergy of both APTES and PDA resulted in the formation of PDA–silanol bond, which was denoted by the formation of crystals on the surface of the rougher Fibers A possible mechanism for the reactive synergy of PDA and APTES is illustrated in Figure 14. Table 4 summarizes selective literatures on the preparation, modification, and morphology of natural fiber-reinforced PBS.

**Figure 15:**
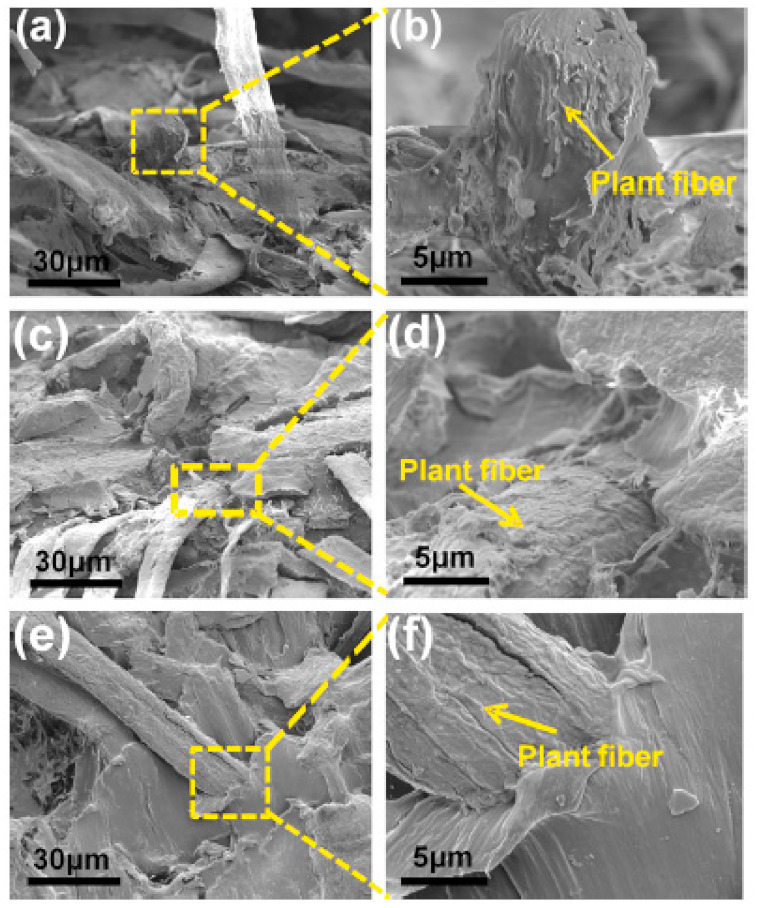
SEM of waste paper (WP)/PBS composites at various WP contents after flexural fracture: (**a**) and (**b**) 10 wt.% WP, (**c**) and (**d**) 30 wt.% WP, and (**e**) and (**f**) 60 wt.% WP [125]. Copyrights obtained from Elsevier.

**Figure 16:**
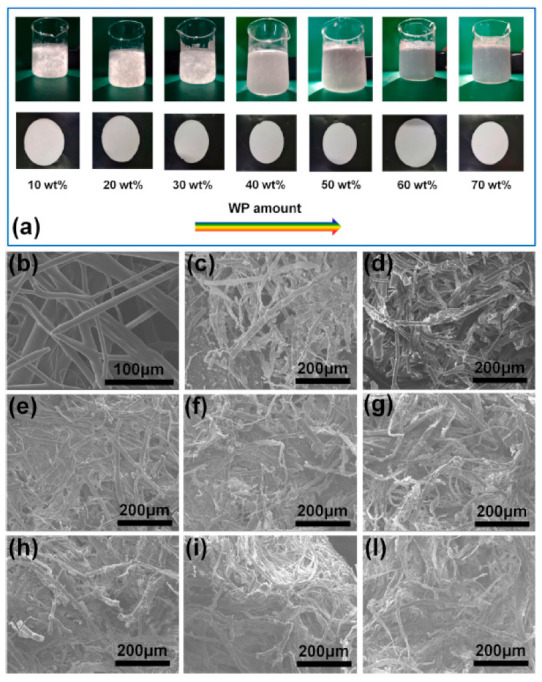
(**a**) Photographs of WP/PBS pulp suspension. SEM images of WP/PBS composites at various WP contents showing the distribution of the plant fibers in WP are shown in: (**b**) pure PBS, (**c**) pure WP, (**d**) 10 wt%, (**e**) 20 wt% WP, (**f**) 30 wt% WP, (**g**) 40 wt% WP, (**h**) 50 wt% WP, (**i**) 60 wt% WP and (**l**) 70 wt% WP [125]. Copyrights obtained from Elsevier.

Zhao et al. [125] modified waste paper (WP) in WP/PBS composites by immersion in a *γ*-methacryloxypropyl trimethoxy silane (KH570) solution. The WP/PBS composites were prepared from ultrafine PBS fibers and WP via a paper manufacturing and compression molding method. Scanning electron microscopy (SEM) images of fractured composite samples showed nearly no defects on the fractured samples at various WP contents. The SEM images also revealed that large portions of PBS were still attached to the plant fibers from WP even after fracture (see Figure 15). This was an indication of a strong interfacial interaction between the plant fibers and PBS. When WP was treated with KH570, the plant fibers in WP generated stable chemical bonds. Therefore, the surface energy of the plant fibers and interfacial tension between the plant fibers and PBS were reduced, resulting in less cohesion among plant Fibers The plant fibers were easily dispersed in the PBS matrix due to their compatibility with PBS, as shown in Figure 16.

The interfacial adhesion between the natural fiber and polymer matrix occurs via two interactions viz adsorption and diffusion, which are governed by Van der Waals forces and hydrogen bonding [120]. In adsorption, both the fiber and matrix are in close contact with each other [120]. This is governed by penetration and proper spreading of both components [120]. Contrastingly, the interdiffusion of both fiber and matrix molecules is governed by improvements in wettability [120]. In summary, based on the above modifications (silanization and alkalination), one can conclude that the alkaline treatment of fibers improves their chemical interaction with the hydrophobic polyester matrix by: (i) enhancing the surface roughness and, as a result, improving the mechanical interlocking; (ii) enhancing the reaction sites with the polyester matrix by uncovering cellulose in the fiber surface. However, in the case of silane treatment, it is evident the silane molecules are bifunctional, whereby one end of the reactive group reacts with the cellulose and the other end of the reactive group reacts with the hydrophobic polyester matrix. The chemical interaction between the fiber and matrix occurs through a siloxane bond.

## 7. Mechanical Properties of PBS/Natural Fiber Biocomposites

**Figure 17:**
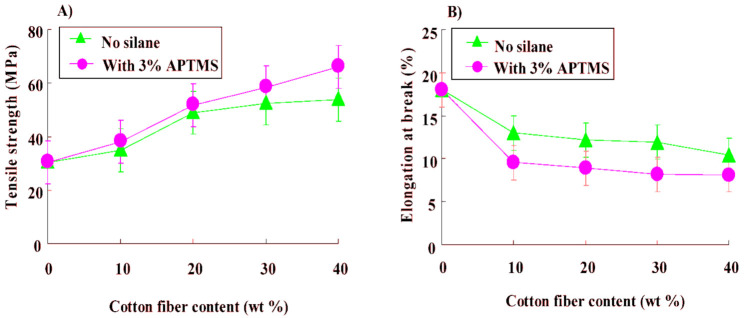
(**a**) Tensile strength and (**b**) elongation at break for PBS/cotton fiber (treated and untreated) [127]. Reprinted with permission from the publisher. Open access.

**Figure 18:**
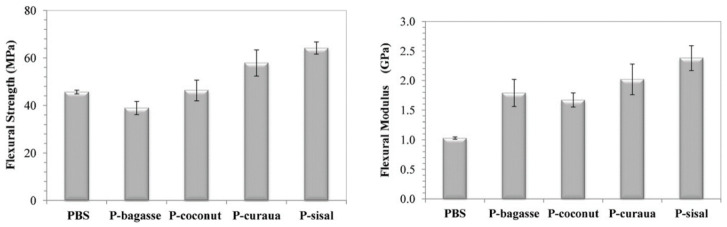
Flexural strength and modulus of PBS and its natural fiber-reinforced composites. Reprinted with permission from the publisher [131].

Natural fibers biocomposites are used in different applications, with majority of these applications depending heavily on the mechanical properties of the resultant biocomposites. Mechanical properties of natural fibers incorporated into PBS matrix were found to be affected by factors such as the type of natural fiber [124,131], modification of the fiber [127,128,133], the type of modifier [127,128,133], the fiber to polymer weight ratios and the fabrication methods [124,127,128,131,133]. In an investigation based on the effect of the type of silane treatment and content on the mechanical properties of PBS/cotton fiber composites, cotton fiber was treated with three types of silane, i.e., 3-aminopropyltriethoxysilane (APTES), 3-aminopropyltrimethoxysilane (APTMS), N1-3-trimethoxysilylpropyldiethylene triamine (TMSPDET) and 10 wt.% of cotton fiber was incorporated into the PBS matrix. Generally, the tensile strength of the treated fiber composites showed higher tensile strength values when compared with neat PBS matrix. It was also observed that the tensile strength of the composites increased with the concentration of the modifiers, except for APTES at 3% concentration, where it was reported to be decreasing. The decrease in tensile strength at 3% APTES was ascribed as an optimum saturation concentration, whereby the ether linkage between the hydroxy groups of the fiber and silane was not formed effectively. APTMS as a modifier showed higher tensile strength than both the TMSPDET and APTES in all investigated concentrations of the silane coupling agent. This was due to the presence of the methoxy groups in the APTMS, which are able to hydrolyze faster than the ethoxy groups in the APTES, with the ethoxy groups inhibiting the hydrolyses into the hydroxyl groups, making it difficult for the formation of the coupling reaction between the fiber and PBS in the presence of APTES. The effect of 3% APTMS on the mechanical properties was further investigated in the same system with higher concentrations of the fiber, i.e., 10, 20, 30 and 40 wt.%. The untreated fiber improved the tensile strength of the composites (Figure 17) by 15%, 62%, 73% and 78% for 10, 20, 30 and 40 wt.% of the fiber, respectively. The tensile strength was further enhanced (Figure 18) by 25%, 71%, 92% and 118% for treated fiber, with fiber contents of 10, 20, 30 and 40 wt.%, respectively. This was due to an enhanced interfacial interaction between the polymer matrix and fiber in the presence of the silane coupling agent. The incorporation of the untreated and treated fiber decreased the elongation at break due to a reduction in the mobility of PBS chains in the presence of the fiber. This is because the incorporation of stiffer material, such as fibers, will decrease the content of PBS in the system, which will result in less PBS being available to elongate, consequently reducing the elongation at break [127].

Nam and co-workers investigated the mechanical properties of the jute fiber-reinforced PBS composites focusing on the: effect of fiber content, soaking time for fiber treatment and alkali surface treatment of the fiber [135]. The jute fiber/PBS composites were fabricated by the compression molding method, with the content of the fiber ranging from 0 to 60 wt.%. There was an enhancement in mechanical properties in the form of tensile strength as well as flexural strength with the content of fiber as far as 50 wt.%, with 60 wt.% of the fiber decreasing the properties. The increase in mechanical properties of the composites was due to a higher strength and modulus of the fiber when compared with the polymer matrix, while the reduction in mechanical properties at the contents of the fiber above 50 wt.% was ascribed to a reduction in wettability due to less PBS content in the composite. The effect of soaking time was investigated with the PBS/jute fiber composite containing 30 wt.% of the fiber. The soaking time of the alkali-treated jute/PBS composites was investigated in the range of 1–6 h, with both the tensile strength and modulus increasing with increasing time from 1 to 3 h; above 3 h, the mechanical properties decrease. The 3-h soaking time was found to be the optimum time for soaking jute fiber because of better mechanical properties when compared with other alkali-treated Fibers The increase in soaking time beyond 3 h had a negative impact on the stress transfer between the PBS matrix and fiber. The increase in soaking time will allow the alkaline treatment enough time to produce brittle as well as rigid fibers which may undergo breakage, inhibiting the fibers from taking part in stress transfer of the composite system, thus reducing the mechanical properties. The effect of alkaline treatment (AT), silane treatment and the synergistic treatment between silane and alkali modifications were investigated. The synergistic treatment between silane and alkali treatment of the fiber resulted in a better mechanical property than the alkaline and silane treated fiber at 50 wt.% fiber content, due to a better interfacial adhesion between the fiber and the matrix. It has been proven in the literature [131] that the type of a natural fiber had an impact on the overall mechanical properties of the natural fiber/PBS composites. In this study, PBS was reinforced with several lignocellulosic fibers such as curaua, sugarcane bagasse, sisal, and coconut. It was reported that both sugarcane bagasse and coconut fiber showed inferior mechanical properties when compared with sisal and curaua, in reference to both flexural strength and modulus (Figure 18). Sugarcane bagasse and coconut fibers showed flexural strengths of approximately 40 and 50 MPa whilst those of sisal and caraua fibers were approximately 70 and 60 MPa. Likewise, the flexural moduli of sugarcane bagasse and coconut fibers were approximately 1.53 and 1.51 GPa, whilst those of sisal and caraua fibers were approximately 2.4 and 2.0 GPa. According to the chemical analysis, it was reported that both the sugarcane bagasse and coconut contained a higher lignin content, which suggested that they are both aromatic, thus reducing their compatibility with an aliphatic polymer matrix. Further, the reduction in flexural strength as well as modulus was ascribed to a weak mechanical bonding between PBS and lignocellulosic fibers (viz sugarcane bagasse and coconut). The surface roughness of both sisal and curaua enhanced their adhesion with PBS matrix, consequently improving their properties (viz flexural strength and modulus). Table 5 summarizes the comparison of the selected studies on the optimum content of the fiber on the mechanical properties of the PBS/natural fiber composites.

## 8. Biodegradation of PBS/Natural Fiber Composites

**Figure 19:**
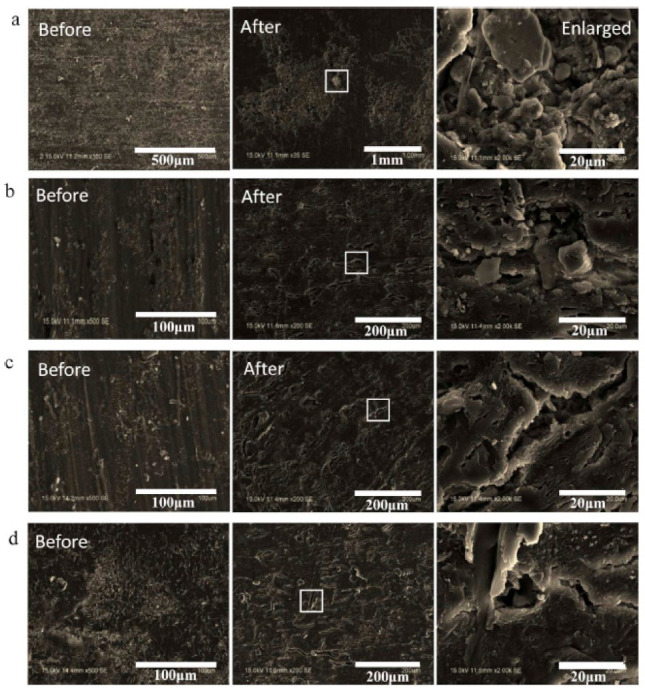
SEM images of pure PBS and sugarcane rind fiber (SRF)/PBS composites at various SRF contents before and after 100 days of soil burial: (**a**) pure PBS, (**b**) 5 wt.% SRF, (**c**) 10 wt.% SRF and (**d**) 15 wt.% SRF [143]. Copyrights obtained from Elsevier.

Few studies have investigated the biodegradation of PBS/natural fibers biocomposites. PBS is biodegradable in various environments such as in lipase solution, soil burial, water, activated sludge and compost [24]. In a study on the biodegradation of different polyesters in sea water via lab and field tests, PBS was found to exhibit slow biodegradation in the BOD (biochemical oxygen demand) test using seawater. However, in the field test, PBS exhibited minimal weight loss when immersed in seawater. The differences in degradation behaviour were attributed to differences in the variety of life in the sea, and the physical as well as chemical defects derived from the environment [140]. Furthermore, a study on the biodegradability of different aliphatic polyesters such as poly(3-caprolactone), poly(*β*-hydroxybutyrate/valerate) and PBS under marine conditions revealed that the degradation of PBS was minimal as compared with other polymers [141]. In another study, the percentage biodegradation of pure PBS in seawater was very low even after 28 days [142]. From the literature, it is clear that PBS exhibits poor degradation in marine environments [42]. Since the addition of certain fillers has been found to improve the degradation of PBS [42], more studies need to be conducted on the role of fillers to enhance the degradation of PBS under marine conditions. The incorporation of natural fiber fillers into have been found to enhance the degradation of PBS [42]. For example, a study on the influence of sugarcane rind fiber (SRF) on the biodegradability of PBS/SRF composites was carried out under natural soil conditions. The results showed that the incorporation of SRF accelerated the degradation of PBS. The enhanced degradation was attributed to the hydrolysis of amorphous regions in PBS, which facilitated the activity of microorganisms [143]. Figure 19 below shows the surface morphologies of SRF/PBS composites before and after soil burial for 100 days. The surfaces of the samples were smooth before soil burial. However, a significant number of pores and grooves were observed on the surface of the samples after soil burial for 100 days. This was an indication that the samples were eroded by the microorganisms during the soil burial process. The enlarged images show that the composites had a higher degree of erosion than the pure PBS. Fiber peeling and breakage were observed on the surfaces of composites of the composites. Pores were also observed around the Fibers This was an indication that the hydrophilic group of SRF absorbed more moisture and microorganisms, which aggravated the erosion effect [143].

**Figure 20:**
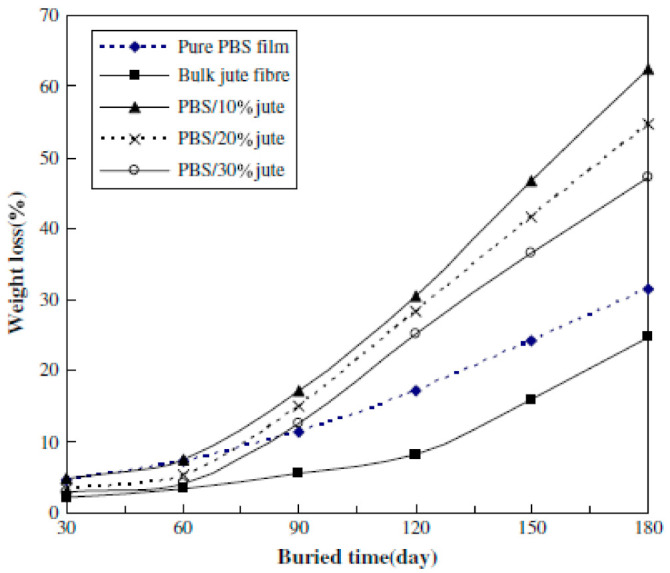
Weight loss of buried pure PBS film, bulk jute fiber and PBS/jute fiber composites at various fiber contents [103]. Elsevier.

**Figure 21:**
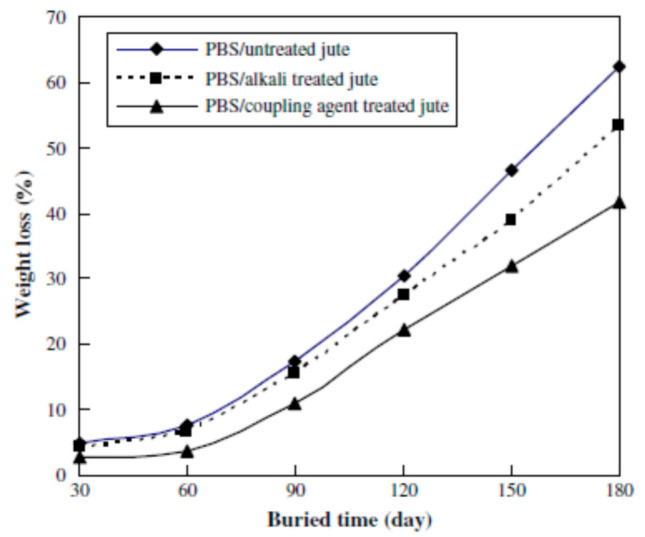
Weight loss of PBS/jute fiber composites consisting of untreated, alkali- and coupling agent-treated jute fibers [103]. Elsevier.

In another study, the incorporation of jute fibers was found to enhance the biodegradation of PBS in PBS/jute fiber composites buried in compost soil [103], as shown in Figure 20. In Figure 20, the weight losses of the PBS/jute fiber composites are higher than those of pure PBS film and bulk jute fiber. The weight losses of the composites also decreased with increasing fiber content. In the same study, it was also reported that the surface modification of fibers had an influence on the biodegradability of jute Fibers Alkali and coupling agent treatments were introduced to modify the properties of jute fibers, and composites reinforced with the treated fibers were subsequently prepared. Results showed that the weight loss of the PBS/jute fiber composites consisting of treated jute fibers decreased after burial in compost soil (this illustrated by Figure 21). The decrease in weight loss was attributed to improved interfacial adhesion between the treated fibers and the PBS matrix [103].

## 9. Specific Applications of the Natural Fiber-Reinforced PBS Composites and Their Natural Fiber PBS Blend Composites

**Figure 22:**
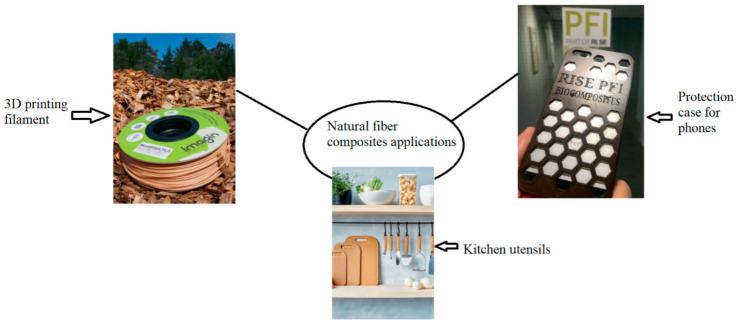
Selective applications of natural fiber composites. Reprinted with permission from the publisher [145].

Generally, natural fibers incorporated in polymer matrices have been employed as replacements for metal-based composites in different industrial applications including marine, aerospace, and sporting goods. The automotive industry has been the leading industry in terms of the utilization of the natural fiber-reinforced biopolymer composites. For example, in Europe it was reported that both wood and cotton were utilized in industry at rates of 38% and 25%, respectively, by the year 2012 [144]. The second leading industry, in terms of the usage of natural fiber-reinforced biopolymer composites, is the construction industry. The main reason for an emerging usage of natural fiber biocomposites in building applications is the global demand for utilization of eco-friendly materials. Figure 22 summarizes selective applications of natural fiber composites.

Poly(butylene succinate) (PBS) applications have been reported mostly with its blend with polylactic acid (PLA). PBS/PLA blends have been used in biomedicine, agriculture, and food packaging [146]. Specific applications of the natural fiber-reinforced PBS blends are shown in Table 6.

## 10. Conclusions and Future Remarks

Natural fibers, incorporated in biopolymers to form biocomposites, have emerged as an attractive material to replace non-biodegradable materials to solve the persisting problem of non-biodegradable plastics waste. The automotive industry is the leading industry in terms of utilizing environmentally friendly natural fiber/biopolymer composites to: (i) enhance fuel efficiency, (ii) reduce energy consumption, and (iii) exert a positive impact on the environment. However, for natural fiber bio-composites to be used in different applications, certain properties must be met. The review discussed a broad understanding of the properties of PBS/natural fiber composites for advanced applications. A major aspect, which plays a critical role in the overall properties of the fiber and polymer, is the weak interfacial interaction between the fiber and PBS matrix. A lot of methods have been employed to modify the fibers, including: (i) physical, (ii) chemical, and (iii) physicochemical treatments. Amongst the above-mentioned methods, chemical methods, especially alkaline treatment, seems to be the most utilized method for reinforced fiber modification in PBS matrices. This method is preferred because it can enhance the surface roughness of the fibers and improve the overall interaction between the fiber and polymer matrix. Generally, it has been reported that the modified composites showed better mechanical properties when compared with unmodified bio-composites. However, there is a growing concern about the utilization of chemical modification method for fiber modification due to the type of solvents that are involved in the process. Going forward, there is a need to try and find more environmentally friendly methods for fiber modification. Furthermore, it is well documented in this review paper that natural fiber/PBS biocomposites are promising materials in terms of reducing the emission of pollutants, and increasing energy recovery and biodegradability. However, despite the advantages of the natural fiber/PBS system, both the natural fiber and PBS are highly flammable, and, to our knowledge, limited studies have investigated the flammability properties of the PBS/natural biocomposites. Another aspect of the PBS/natural fiber composites that is worth studying is the natural fiber/PBS hybrid composites. A combination of two fibers in a single matrix provides properties that cannot be obtained by a single fiber, which will widen the applications of the PBS/natural fiber composites. Most of the natural fibers used in PBS biocomposites are plant based. Therefore, more studies need to be conducted on PBS composites reinforced with animal-based natural Fibers Animal-based natural fibers such as silk are already used in biomedical applications as suture threads and they show great biocompatibility with human cells. Biomedical applications such as tissue engineering and wound dressing require the use of porous composite scaffolds. The porous nature of the scaffolds promotes cell attachment and growth, which facilitates nutrient and oxygen diffusion. Therefore, the design of porous PBS/animal-based natural fiber composite scaffolds may have a future in tissue engineering and wound dressing applications.

## Figures and Tables

**Table 1:** World production per year of natural fibers [16,18,19]. Copyrights with permission from Elsevier [19].

Type of Fiber	World Production (10^3^ Ton) per Year
Abaca	70.00
Bamboo	30,000.00
Caraua	>1.00
Coir	100.00
Cotton	25,000.00
Flax	830.00
Grass	700.00
Hemp	214.00
Jute	2300.00
Kenaf	970.00
Oil palm	40.00
Pineapple	74.00
Ramie	100.00
Sisal	378.00
Sugar cane bagasse	75,000.00

**Table 2:** Physical properties of PBS as compared with those of polyolefins such as polypropylene (PP), high density polyethylene (HDPE) and low-density polyethylene (LDPE) [24]. Copyrights obtained with permission from Wiley.

Physical Properties	PBS	PP	HDPE	LDPE
Glass transition temperature (°C)	−32	−5	−120	−120
Melting temperature (°C)	114	163	129	110
Heat distortion temperature (°C)	97	110	82	49
Tensile strength (MPa)	34	33	28	10
Elongation at break (%)	560	415	700	300
Izod impact strength (J/m)	300	20	40	>400
Degree of crystallinity (%)	35–45	56	69	49

**Table 3:** Chemical composition of various natural Fibers

Natural Fiber	Cellulose Content (%)	Hemicellulose Content (%)	Lignin Content (%)	Pectin Content (%)	Wax Content (%)	Ash Content (%)	Moisture Content (%)	Refs
Abaca	56–63	20–25	7–12	0.8	3	-	-	[19,59,60,61,62,63,64]
Acacia Arabica (Indian gum Arabic tree)	68.10	9.36	16.86	-	0.49	-	-	[65]
Acacia Leucophloea (White-barked acacia)	68.09	13.6	17.73	-	0.55	0.08	8.83	[65,66]
Acacia Planifrons (Umbrella thorn)	73.1	9.41	12.04	-	0.57	4.06	8.21	[65]
Agave	68.42	4.85	4.85	-	0.26	-	7.69	[65]
Alfa	45.4	38.5	14.9	-	2	-	-	[59,60,61,63,64]
Areca	57.35–58.21	13–15.42	23–24	-	0.12	-	-	[63,67,68]
Bagasse	32–55.2	16.8–25	19–25.3	10	-	-	-	[52,59,60,63,64,69,70]
Bamboo	26–55	20.5–30	15–32.2	-	-	-	-	[19,49,52,59,60,63,64,71,72]
Banana	60–65	12.5–25	5–10	4	-	-	-	[52,59,63,64,69,70,73,74,75,76]
Barley	31–45	27–38	8–19	-	2–7	-	-	[52,63,73,77]
Cissus Quadrangularis (veld grape) root	77.17	11.02	10.45	-	0.14	-	7.3	[65]
Cissus Quadrangularis (veld grape) stem	82.73	7.96	11.27	-	0.18	-	6.6	[65,78]
Coir	32–45.6	0.15–21	40–45	4	-	-	-	[52,57,59,60,61,63,64,79,80,81]
Corn	38–40	28	7–21	-	3.6–7	-	-	[63,73]
Cotton	82.7–90	4–5.7	0.75	6	0.6	-	-	[59,60,61,63,64]
Curaua	70.7–73.6	9.9	7.5–11.1	-	-	-	-	[19,59,60,61,63,64,69]
Dichrostachys Cinerea (sicklebush)	72.4	13.08	16.89	-	0.57	3.97	9.82	[65,82]
Epipremnum Aureum (Devil’s ivy)	66.34	13.42	14.01	-	0.37	4.61	7.41	[65,83]
Eucalyptus	41.7	32.56	25.4	8.2	0.22	-	-	[63,84]
Flax	62–81	4–20.6	2.2–5	0.9	1.5–1.7	-	10	[19,52,59,60,63,64,65,69,70,85]
Furcraea Foetida	68.35	11.46	12.32	-	0.24	6.53	5.43	[65]
Hemp	67–81	5.5–22	2.9–13	0.8–0.9	0.8–2.3	-	10.8	[19,52,59,60,63,64,65,86,87]
Henequen	60–77.6	28	8–13.1	-	0.5	-	-	[59,60,61,63,64]
Heteropogon Contortus (Spear grass)	64.84	19.34	13.56	-	0.22	-	7.4	[65,88]
Hibiscus	28	25	22.7	-	-	-	-	[63,89]
Isora	74	-	23	-	1.1	-	-	[59,60,63,64]
Jute	56–72	12–35	9–14	0.2	0.5	1	12.6	[19,52,57,59,60,63,64,65,70,90,91,92]
Kenaf	53.14–53.5	3–33	8.18–21.5	2	-	3.5	9	[52,59,60,63,64,65,76,79,93]
Kudzu	33	11.6	14	-	-	-	-	[59,64]
Nettle	86	10	-	-	4	-	-	[59,64]
Oil Palm	65	-	29	-	-	-	-	[59,64]
Palm	32–35.8	24.1–28.1	26.5–28.9	-	-	-	-	[52,94]
Perotis indica (Indian comet grass)	68.4	15.7	8.35	-	0.32	4.32	9.54	[65,95]
Phromium	67	30	11	-	-	-	-	[60,63]
Piassava	28.6	25.8	45	-	-	-	-	[59,64]
Pine	67.29	67.29	11.57	-	-	-	-	[52,96]
Pineapple	80.5-81	17.5	8.3–12.7	4	-	-	-	[19,59,60,63,64]
Prosopis Juliflora (Mesquite)	61.65	16.14	17.11	-	0.61	5.2	9.48	[65]
Ramie	72	5–16.7	0.6–0.8	2	0.3	-	-	[59,60,63,64,86]
Red Banana Penduncle	72.90	11.01	15.99	-	0.32	2.79	9.36	[65]
Rice	59.9	59.9	20.6	-	-	-	-	[52,97]
Rice husk	28–36	23–28	12–14	-	14–20	-	-	[63,89]
Sida Rhombifolia (arrowleaf sida) stem	75.09	15.43	7.48	-	0.49	4.07	12.02	[65,98]
Sisal	57–73	11.5–16	8–12	1.2	2	-	17	[19,52,59,60,63,64,65,72,99,100]
Sorghum	27	25	11	-	-	-	-	[63,73]
Sponge Gourd	63	19.4	11.2	-	3	-	-	[59,64]
Straw (wheat)	38–45	15–31	12–20	-	-	-	-	[59,64]
Sun Hemp	41–48	8.3–13	22.7	-	-	-	-	[59,64]
Water Hyacith	43.58–47.38	19.77–22.23	9.52–13.08	-	-	-	-	[52,56,57,85]
Wheat	30–38	26–50	15–19	-	6.8	-	-	[52,63,77,84]

-: means not reported.

**Table 4:** Summary of selective studies on the preparation, modification and morphology of natural fiber-reinforced PBS.

PBS/Natural Fiber Composites	Preparation Methods	Modification	Summary of the Results	Refs
PBS/alfa fiber	Compression molding method	Alkaline treatment	Fiber alkaline treatment resulted in: - Removal of pectin, lignin, waxes and hemicellulose. - Improved interfacial adhesion. - Fibers embedded into PBS matrix.	[126]
PBS/Cotton fiber	Compression molding at 150 °C for 5 min	Silane treatment	- Poor interfacial interaction in the absence of fiber treatment. - Improved interfacial adhesion due to fiber treatment.	[127]
PBS/rice straw fiber	Injection molding	Amino silane treated	Treatment of fibers with 3-(2-aminoethylaminopropyl)-trimethoxysilane (AEAPTMES) achieved the best interfacial bonding results.	[128]
PBS/Kenaf fiber (KF)	Melt mixing method	----------------	Fibers were embedded in the PBS matrix; however, there was a phase separation between kenaf and PBS.	[129]
PBS/Cellulose fiber (Cellulose extracted from kenaf fiber and commercial cellulose)	Melt mixing at 200 °C for 5 min	Hydrochloric acid (HCl) and sodium hydroxide (NaOH) treatment	- The morphology of the PBS/KTH, PBS/EC, and PBS/CC composites were reported. - In this study, KTH denotes kenaf treated with HCl, EC is the extracted cellulose fiber and CC stands for commercial cellulose. - All composites showed visible gaps between the fiber and matrix, due to poor interaction.	[130]
PBS/lignocellulosic fibers (coconut, sugarcane bagasse, curaua, sisal)	Thermo-pressing mold method	Ethanol and cyclohexane extraction process	- All composites, to some extent, showed voids and fiber pull outs; however, a better interaction between the two phases was reported. - The surface roughness of the curaua and sisal fibers promoted mechanical bonding between PBS and Fibers	[131]
PBS/ramie fiber Fabric (RFF)	Thermal compressive process	Treatment with 2% of 3 Triethoxysilylpropylamine (KH550)	- KH550 treated fibers were embedded into the PBS matrix due to improved interfacial bonding.	[132]
PBS/curaua fibers	Compression molding	Enzymatic treatment	- Homogenous distribution of untreated and treated fibers in the PBS matrix at 10 wt.% fiber loading. - Strong adhesion between the fiber and polymer matrix with no voids.	[133]
PBS/bamboo fiber	Hot press molding	Acetoxylation	- Presence of strong van der Waals and hydrogen-bond interactions as well as physical adsorption between functional groups of composites. - Tensile strength and the initial decomposition temperature of Acetylated-BF/PBS were improved by 18% and 12.2 °C, respectively, due to the fiber modification	[134]

**Table 5:** Comparison of the selective studies on the optimum content of the fiber on the mechanical properties of the PBS/natural fiber composites.

PBS/Natural Fiber	Preparation Method	Optimum Concentration (%) of the Fiber	Type of Modifier	Mechanical Properties	Refs
PBS/Palm fiber (PF) and glycidyl methacrylate-grafted poly(butylene succinate) (PBS-g-GMA)/PF	Melt mixing at 140–150 °C	40 wt.% for modified fiber composites.	Glycidyl methacrylate-grafting	- Mechanical properties of the PBS-g-GMA/PF system was higher than that of PBS/PF composites due to a better adhesion between PF and PBS-g-GMA matrix.	[136]
PBS/Ramie fibers	Twin-screw extrusion and injection molding	30% of the ramie content.	Single ramie fiber was modified with silane, alkali, acetic anhydride, and maleic anhydride treatment.	- The interfacial adhesion between the polymer and fiber was done through interfacial shear strength (IFSS). - It was found that alkali treatment showed the highest IFSS when compared with other modifiers. - Improved tensile strength and modulus of PBS with fiber loading (untreated and treated fiber). - Mechanical properties of treated fiber composites were better than those of untreated fiber composites.	[137]
PBS/rice straw fiber	Injection molding	30% of silane treated fibers	3-aminopropyltriethoxysilane (APTES), 3-aminopropyltrimethoxysilane (APTMES), 3-(2-aminoethylaminopropyl)-triethoxysilane (AEAPTES) and 3-(2-aminoethylaminopropyl)-trimethoxysilane (AEAPTMES) fiber treatment.	- Mechanical properties were investigated at 70:30 of polymer: fiber (with fiber size of 100–300 µm). - Improved tensile strength due to fiber treatment. Best results were obtained with AEAPTMES fiber treatment.	[128]
PBS/curaua fiber	Thermo-pressing molding	30 wt.% of the curaua fiber.	Alcohol and cyclohexane fiber pre-treatment.	- Enhancement in impact resistance with fiber loading. Impact resistance increased from 52 Jm^−1^ (for PBS) to approximately 345 Jm^−1^ (at 30 wt.% fiber loading). - Flexural modulus improved by 64% with fiber loading when compared with neat PBS.	[138]
PBS/oil palm mesocarp fiber (OPMF) and PBS/oil palm empty fruit bunch fiber (OPEFBF)	Melt blending	Various contents of the fiber resulted in improvement of various parameters of mechanical properties.	Fibers were washed with distilled water and acetone.	- Decrease in tensile strength with fiber loading. Tensile strength decreased from 37.10 MPa (for neat PBS) to 25.55 MPa (10 wt% OPMF) and 25.67 MPa (10 wt% OPEFBF).	[139]

**Table 6:** Specific applications of different PBS/natural fibers composites and natural fiber PBS blend composites.

PBS/Natural Fiber or PBS Blend Natural Fiber Composites	Preparation Method	Intended Application	Summary of Results	Refs
Poly(butylene succinate) (PBS)/cellulose nanocrystals (CNC) bio-composite scaffolds	Electrospinning technique	Tissue engineering	- 3 wt.% CNC exhibited improved bio-degradability from 4.5 wt.% (for neat PBS) to 13.47 wt.% (for PBS/CNC scaffolds) after 28 days. - Improved proliferation on the PBS/CNC scaffolds with 3 wt.% CNC as compared to neat PBS scaffolds after 7 days.	[147]
Poly(butylene succinate)/date palm fibers (DPF) biocomposites	Injection molding process	Green and sustainable products	- Improved rigidity enzymatically treated Fibers Rigidity increased by approximately 29% (for palm fibers) and 42% (for trunk fibers).	[148]
Polybutylene-succinate-modified Tapioca starch blend/Empty fruit brunch (EFB) composite films	Hot press technique	Agricultural munch films	- Decrease in tensile and flexural strengths with fiber loading. - Increased rate of water vapour permeability at higher EFB fiber contents. - Little or no changes in the thermal properties with EFB incorporation.	[149]
Poly(lactic acid)/poly(butylene succinate)/cellulose fiber composite foams	Twin screw extrusion	Hot cups packaging	- Decreased viscosity with cellulose fiber loading. - All composites exhibited a shear thinning behavior which was attributed to the orientation of the fibers towards the flow direction. - A low cellulose fiber content resulted in a lower melt viscosity at all shear rates. - Tensile strength, tensile modulus and percentage elongation at break increased with increasing cellulose fiber content. - Degree of crystallinity increased with incorporation of cellulose Fibers - PLA/PBS/CF composites exhibited improved thermal degrading properties as compared to PLA.	[150]
Poly(butylene succinate)-Poly(lactic acid) blend/wood flour	Hot melt blending and hot pressing	Used in diverse commercial applications	- PBS/PLA and wood flour were not compatible due to poor wettability and interfacial adhesion. Incorporation of Fusebond MB 100 D improved the interfacial bonding. - The addition of Fusebond MB 100 D improved the tensile and impact strengths under high dynamic loading.	[151]
Poly(butylene succinate)/hemp fiber composites	Film stacking compression molding method followed by extrusion and injection molding	Low cost composite materials	- Tensile and flexural properties increased whilst impact strength decreased with increasing fiber content. - The surface modification of fibers did not affect the mechanical properties of the composites but the fiber–matrix adhesion and resistance to moisture absorption were improved.	[152]
Lignin/poly(butylene succinate) composites	Hot melt extrusion	Biomedical applications	- The composites exhibited excellent antioxidant activity. - The composites resisted the adherence of *staphylococcus aureus*.	[153]
Sustainable tetra pak recycled cellulose (rCell)/poly(butylene succinate) woody-like composites	Melt compounding using a Brabender mixer	Building materials, furniture and food packaging	- Incorporation of 50 wt.% rCell into PBS improved the hardness of the composites. - The rCell composites exhibited a higher Young’s modulus compared to pristine PBS. - PBS/rCell composites were biodegradable in soil under composting conditions.	[154]
Poly(butylene succinate)-isora nanofibil (PBS-INF) composites	Melt mixing of PBS with different loadings of INF	Automotive interior and food packaging	The incorporation of INF had a positive influence on the thermo-physical properties of PBS.	[155]
Poly(butylene succinate)/microcrystalline cellulose (MCC)/nanofibrillated cellulose (NFC) sustainable polymer composites	Melt mixing in a Brabender at 140 °C and rotation speed of 70 rpm	Packaging, medicine, automotive, construction, sustainable housing	- Young’s modulus and the storage modulus of the composites increased by about two-fold at 20 °C. - Thermal degradation temperature of the composites increased by 60 °C compared to that of MCC and NFC. - The decomposition of PBS under composting conditions was up to 75 days. However, the incorporation of MCC/NFC resulted in a decomposition of up to 60 days.	[156]
Poly(butylene succinate)/cellulose nanocrystals (PBS/CNCs) composite scaffolds.	Supercritical carbon dioxide (Sc-CO_2_) foaming process	Tissue engineering	- Scaffolds with a well-defined bimodal open pore structure were obtained. The porous was composed of pores with diameters of 11.0 and 68.9 μm as well as a porosity 95.2%. - Scaffolds exhibited improved mechanical compressive strength, hydrophilicity, rate of in vitro degradation and compatibility.	[157]

## Data Availability

Not applicable.
